# Differential Expression of NK Receptors CD94 and NKG2A by T Cells in Rheumatoid Arthritis Patients in Remission Compared to Active Disease

**DOI:** 10.1371/journal.pone.0027182

**Published:** 2011-11-15

**Authors:** Ceara E. Walsh, Elizabeth J. Ryan, Cliona O’Farrelly, Lucy Golden-Mason, Oliver FitzGerald, Douglas J. Veale, Barry Bresnihan, Ursula Fearon

**Affiliations:** 1 Translation Rheumatology Research Group, Dublin Academic Medical Centre, St. Vincent's University Hospital, Elm Park, Dublin, Ireland; 2 School of Biochemistry & Immunology, Trinity College, Dublin, Ireland; Karolinska Institutet, Sweden

## Abstract

**Objective:**

TNF inhibitors (TNFi) have revolutionised the treatment of rheumatoid arthritis (RA). Natural killer (NK) cells and Natural Killer Cell Receptor^+^ T (NKT) cells comprise important effector lymphocytes whose activity is tightly regulated through surface NK receptors (NKRs). Dysregulation of NKRs in patients with autoimmune diseases has been shown, however little is known regarding NKRs expression in patients with TNFi-induced remission and in those who maintain remission vs disease flare following TNFi withdrawal.

**Methods:**

Patients with RA were recruited for this study, (i) RA patients in clinical remission following a minimum of one year of TNFi therapy (n = −15); (2) Active RA patients, not currently or ever receiving TNFi (n = 18); and healthy control volunteers (n = 15). Patients in remission were divided into two groups: those who were maintained on TNFi and those who withdrew from TNFi and maintained on DMARDS. All patients underwent full clinical assessment. Peripheral blood mononuclear cells were isolated and NKR (CD94, NKG2A, CD161, CD69, CD57, CD158a, CD158b) expression on T-(CD3^+^CD56^−^), NK-(CD3^−^CD56^+^) and NKT-(CD3^+^CD56^+^) cells was determined by flow cytometry.

**Results:**

Following TNFi withdrawal, percentages and numbers of circulating T cells, NK cells or NKT cell populations were unchanged in patients in remission versus active RA or HCs. Expression of the NKRs CD161, CD57, CD94 and NKG2A was significantly increased on CD3^+^CD56-T cells from patients in remission compared to active RA (p<0.05). CD3^+^CD56-T cell expression of CD94 and NKG2A was significantly increased in patients who remained in remission compared with patients whose disease flared (p<0.05), with no differences observed for CD161 and CD57. CD3^+^CD56^−^ cell expression of NKG2A was inversely related to DAS28 (r = −0.612, p<0.005).

**Conclusion:**

High CD94/NKG2A expression by T cells was demonstrated in remission patients following TNFi therapy compared to active RA, while low CD94/NKG2A were associated with disease flare following withdrawal of therapy.

## Introduction

Rheumatoid arthritis (RA) is the most common form of inflammatory arthritis affecting 1% of the population. Left untreated RA leads to joint deformity and disability [1]. RA is characterised by symmetrical erosive polyarthritis, with extra-articular manifestations in some patients. Activated T cells and innate cells such as macrophages contribute to the development of synovial inflammation by secreting TNFα, a potent pro-inflammatory cytokine [2]. TNFα inhibits both bone formation and proteoglycan synthesis while inducing bone and proteoglycan resorption. It also stimulates metalloproteinase and collagenase production, triggers inflammatory cytokine cascades and increases adhesion molecule expression by infiltrating immune cells. TNF inhibitors (TNFi) improve disease activity indices (clinical and laboratory) and inhibit radiographic progression [3]−[6]. The use of TNFi has revolutionised the treatment of RA patients, particularly in patients with moderate to severe RA [3]−[7].

However, TNFi are expensive and have potential for serious side-effects. Prior to routine use of biologic therapies, the average annual medical cost for a patient with RA was $8500 [8]. Studies have demonstrated mean annual costs of TNFi between $12,146 and $15,617 depending on the agent prescribed. When other expenses are taken into account, e.g. administration in an OPD setting and concomitant disease modifying anti-rheumatic drug (DMARD) use, the cost may rise to $18,046 [9], [10]. An important goal for rheumatologists treating RA patients is to identify markers that can (1) predict response to TNFi, (2) predict remission rates and (3) predict those patients who can maintain remission following withdrawal of TNFi [11], [12].

Natural killer cells (NK), T cells and natural killer T (NKT) cells participate in aetiology and regulation of RA pathogenesis [13]−[16]. NK cells are key players in innate immunity, their primary function killing of virally infected or transformed cells. They can also regulate the adaptive immune response through their ability to produce cytokines. The activity of NK cells is tightly controlled through a variety of stimulatory, co-stimulatory and inhibitory receptors (NKRs) [17]. Dysregulated expression of NKRs and impairment of NK function have been demonstrated in RA. For example, increased expression of the CD94 receptor with concomitant reduction in the expression of inhibitory Killer Ig-Like Receptors (KIR) has been demonstrated in patients with RA [13]. In addition, Richter et al. have recently demonstrated that the NK receptor CD161 contributes to impairment of NK cell cytotoxicity and responsiveness to specific ligands in patients with RA [14].

T cells that co-express NKRs comprise approximately 5−15% of the peripheral T cell pool and have the ability to mediate functions of both T and NK cells. These cells may also express activatory or inhibitory NKRs e.g. CD94/NKG2A (inhibitory) or NKG2D (activatory) and are known as NK receptor^+^ T cells (NKT cells) [18]. Levels of NKR+ T cells are increased in the peripheral blood and synovial tissue of patients with active RA [15], [19]. High levels of IL-15 and TNFα in RA patients contribute to the expansion of the NKG2D^+^ T cell population, cells that may have greater autoreactive potential due to their high expression of activatory receptors [15]. A significant proportion of T cells (CD4^+^CD28^−^) expressing NKG2D display autoreactive responses against RA synovial fibroblasts [15], [20]. Furthermore increased expression of activatory KIR receptor KIR2DS2 on CD4^+^CD28^−^ cells have been shown to correlate with RA disease activity [21].

The aim of this study was to examine NK receptor expression on lymphoid phenotypes in patients in TNFi-induced remission compared to active disease, and to investigate their expression in patients with continued remission compared to disease flare following TNFi withdrawal.

## Methods

### Patient Cohorts

All research was carried out in accordance with the Declaration of Helsinki and approval for this study was granted by the St. Vincent's University Hospital medical research and ethics committee. Rheumatoid arthritis (RA) patients diagnosed according to the criteria of the American College of Rheumatology [22] were recruited from the rheumatology outpatient clinics at St. Vincent's University Hospital (SVUH). This study was approved by the SVUH Medical Research and Ethics Committee; all patients provided fully-informed written consent. Two cohorts of patients with RA were recruited from the Rheumatology clinics, St. Vincent's University Hospital; (1) RA patients in clinical remission 28 joint disease activity score (DAS28) <2.6 for 6 months] (n = 15) following a minimum of one year of TNFi therapy; and (2) Active RA patients, not currently or ever receiving TNFi (n = 18). All patients fulfilled the American College of Rheumatology criteria for RA. Healthy control volunteers were also recruited (n = 15).

The RA remission group were subsequently divided into two groups, those that continued on TNFi therapy or those that were withdrawn from biologic therapy and maintained on substituted optimal DMARD therapy.

Clinical and laboratory assessments were performed in remission patients at baseline, 1, 3 and 6 months. After 6 months, patients who continued TNFi were given the further option of withdrawing and substituting optimal DMARD therapy. Full clinical and laboratory assessments were also performed in the active RA group. Clinical assessment included physical examination and joint counts. Erythrocyte Sedimentation Rate (ESR) and C-reactive protein (CRP) were measured and the DAS28 calculated at each visit. Functional assessment using the health assessment questionnaire-disability index (HAQ-DI) was performed. Short-form−36 (SF-36) data was collected at baseline and 6 months (or study termination, if earlier). RA patients experiencing a disease flare were treated according to best clinical practise with corticosteroids ≤20 mg/day reducing over 2 weeks plus recommencement of TNFi if appropriate.

### Peripheral blood mononuclear cell separation

Fifteen ml of heparinised blood was collected at baseline from each participant and at each clinic visit thereafter from patients in the remission cohort. PBMCs were isolated by density gradient centrifugation (Lymphoprep; Nycomed, West Midlands, UK). Cells were washed in Roswell Park Memorial Institute (RPMI)-1640 (Gibco-BRL, Paisley, UK) supplemented with penicillin (100units/mL; Biosciences), streptomycin (100units/mL; Biosciences) and fungizone (0.25µg/mL; Biosciences) (sRPMI) and cryopreserved (20% Dimethyl Sulphoxide in fetal bovine serum,) for subsequent analysis. It has previously been demonstrated that cryopreservation does not alter immunological characteristics of NK cells [23].

### Flow cytometric analysis of cell surface antigens

Fluorochrome-labelled (fluorescein isothiocyanate (FITC)/phycoerythrin [PE]/peridinin-chlorophyll-protein complex (PerCP)) monoclonal Abs (MAbs) specific for CD3, CD4, CD8, CD56, CD161, CD94, CD158a, CD158b, HLA-DR and CD57 were obtained from BD Biosciences (Oxford, UK). Anti-NKG2A-PE was obtained from Immunotech (Beckman Coulter, Fullerton, CA). PBMCs were defrosted in warmed sRPMI and resuspended at a concentration of 1 million cells per millilitre of sRPMI. PBMCs (2.5×10^5^ cells) were stained for cell surface antigen expression at 4°C in the dark for 30 min. Cells were then washed twice in phosphate buffered saline (PBS) and fixed in 1% paraformaldehyde (PFA) until analysis. Cells were acquired using a BD FACSCalibur instrument (BD Biosciences) and analysed using CellQuest software (BD Biosciences).

### Statistical analysis

Statistical analysis was performed using SPSS version 12.1.0 for Windows. Non-parametric Mann Whitney U-test was used to compare differences between study cohorts and non-parametric Wilcoxon Signed Rank for related samples. Significance was defined as a *p* value <0.05. Correlation coefficients were determined using Spearman's test.

## Results

### Patient demographics

Baseline characteristics of remission and active RA patients cohorts are shown in (Table 1). Fifteen patients in remission were recruited, ten (66%) patients were female with a mean (SEM) age of 50.27 (4.08) years, disease duration of 9.63 (1.96) years, ESR 8.73 (1.38) mm/hr, CRP 6.00 (1.02) mg/L and 12 (80%) patients were rheumatoid factor positive (RF+). Patients were divided into those who were maintained on therapy compared with those withdrawing therapy (Table 2), No demographic differences were demonstrated between the two groups. Nine of fifteen (60%) patients: (female, n (%), 6 (66.67); age, mean years (SEM), 50.0 (5.6); RF+, n (%), 9 (81.81)) continued on TNFi therapy (Table 2). After 6 months, five of these patients consented to withdraw TNFi therapy. Six patients withdrew TNFi therapy initially and five at 6 months, giving a total of 11 TNFi withdrawal patients (female, n (%), 7 (63.64%); age, mean years (SEM), 52.6 (4.1); RF+, n (%), 6 (66.67)). One patient had a flare in disease activity two weeks after TNFi withdrawal, did not respond to corticosteroids and recommenced their TNFi. No significant difference was seen in clinical, functional assessment (HAQ, SF-36) and laboratory markers of inflammation (ESR CRP) at six months in both groups (Table 2).

**Table 1:** Baseline characteristics of patients in remission following TNF inhibitor therapy and in patients with active rheumatoid arthritis.

	Remission Cohort	Active RA Cohort	p value
Female, n (%)	10(66)	12(66.67)	p = ns
Age, mean(sem)	50.27(4.08)	57.00(3.04)	p = ns
Disease duration, mean (sem)	9.63(1.96)	3.72(0.55)	p = (0.004)
Rheumatoid factor +, n (%)	12(80)	16(88.89)	p = ns
ESR(mm/hr), mean(sem)	8.73(1.38)	35.24(4.87)	p = 0.00003
CRP(mg/L), mean (sem)	6.00(1.02)	26.29(6.32)	p = 0.004

*p<0.05 remission cohort significantly different from active RA cohort.

**Table 2:** Clinical, laboratory and functional assessments in patients cohorts maintaining and withdrawing TNF inhibitor therapy.

	Withdrawal of anti-TNF Therapy			Maintain anti-TNF therapy		
	Baseline	3 months	6 months	Baseline	3months	6months
TJC(0–28) mean±sem	0.36±0.15	0.82±0.54	0.91±0.56	0.56±0.00	0.11±0.22	0.44±0.24
SJC (0-28) mean±sem	0.36±0.28	0.64±0.59	0.91±0.58	0.00±0.38	0.22±0.11	0.33±0.24
ESR (mm Hg) mean±sem	9.20±1.83	9.20±1.91	9.20±2.11	8.56±1.84	8.57±2.20	7.89±1.93
CRP (mg/dl) mean±sem	7.36±1.68	8.18±3.02	8.55±3.28	4.33±0.33	4.50±0.47	4.22±0.22
VAS(0-100 mm) mean±sem	13.82±3.56	14.27±5.75	18.91±5.25	15.22±5.30	28.25±7.70*	21.86±5.22
DAS28 mean±sem	1.88±0.18	1.88±0.43	2.02±0.42	1.82±0.27	1.80±0.25	1.94±0.20
HAQ (0–3) mean±sem	0.216±0.000	0.333±0.047	0.296±0.125	0.278±0.062	0.422±0.077	0.393±0.070
SF-36 PCS mean±sem	45.43±2.73		46.14±2.93	45.50±3.34		41.22±3.06
SF-36 MCS mean±sem	56.43±2.3		53.05±3.13	53.50±3.16		50.47±3.46

*p<0.05 vs baseline.

Active RA patients (n = 18; female, n (%) (12 (63.16)) and fifteen healthy controls (12 (80%)) were recruited (Table 1). The mean (SEM) age of the active RA group was 57.00 (3.04) years and healthy controls 41.67 (2.63) years. Clinical characteristics for the RA patients included mean ESR of 35.24±4.87 mm/hr, CRP of 26.29±6.32 mg/L, and disease duration of 3.72±0.55 years. Thirteen (86.67%) were RF+.

### T cell populations in remission patients, active RA and controls

**Figure 1:**
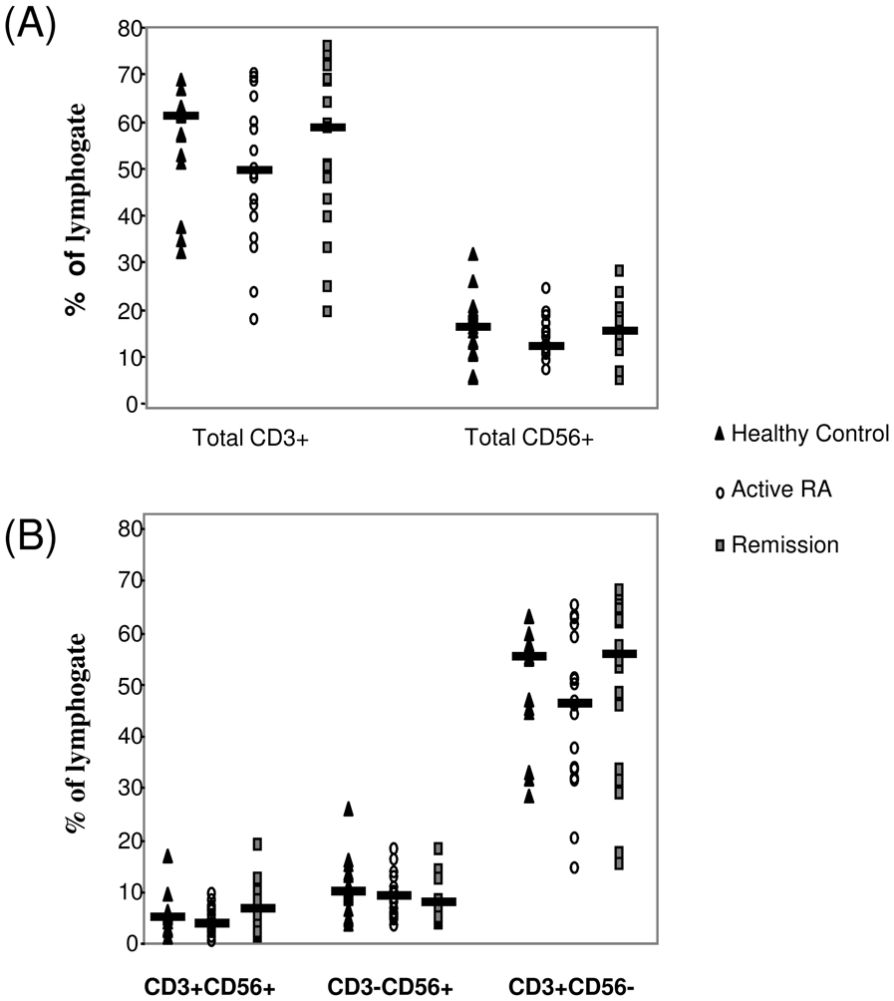
T cell, NK and NKT cell populations in active RA, Remission RA and healthy controls. Graph illustrating T cell, NK and NKT cell populations in healthy controls (N = 15) (▴), patients with active RA (n = 18) (○) and patients in remission following TNF inhibitor (n = 15)(▪). *p<0.05 significantly different.

No difference was demonstrated in percentages of totals of classic T cells or NK cells, (expressed as a percentage of the lymphogates) between patients in remission, patients with active RA or controls (Figure 1A). Furthermore, no significant difference was observed for percentages of NKT(CD3+CD56−), NK (CD3-D56+) or T (CD3+CD56+) cell populations (Figure 1B).

### Expression of NK surface receptors CD161, CD57 and HLA-DR by T cells

**Figure 2:**
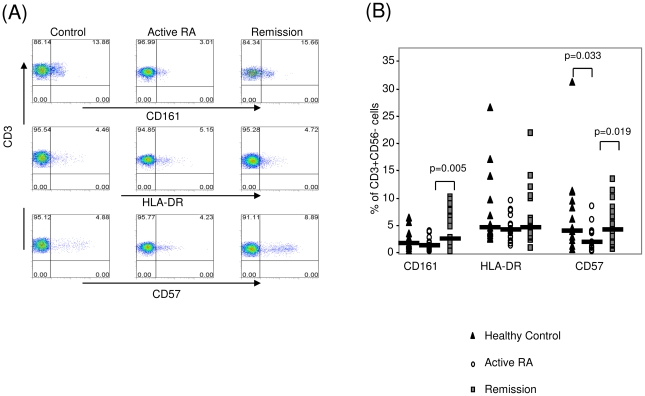
Expression of CD161, HLA-DR and CD57 in active RA, Remission RA and healthy controls. (A) Representative dot plots of CD161, HLA-DR and CD57 expression on healthy control, patient with active RA and patient in remission following TNF inhibitor therapy. (B) CD161, HLA-DR and CD57 expression on CD3+CD56− cells in healthy controls (▴), patients with active RA (○) and patients in remission following TNF inhibitor therapy (▪). *p<0.05 significantly different.

Expression of CD161 by T cells was significantly increased in remission patients (4.11±0.78%) compared to active RA (1.39±0.27%) (p = 0.005) (Figure 2A,B). CD161 expression in remission was not significantly different to levels expressed by the CD3+CD56− cells of the control group (2.28±0.45%) (p = 0.155). Furthermore, an increased proportion of CD3^+^CD56^−^ T cells were CD57^bright^ in both the remission and control groups (5.17±0.88% and 6.71±2.01%) compared to the active RA group (2.57±0.50%) (p = 0.019 and 0.033 respectively) (Figure 2). Expression of HLA-DR on CD3+CD56− cells was similar in remission (6.48±1.24%), active RA (4.49±0.53% (p = 0.406)) and controls (7.40±1.77% (p = 0.873)). Furthermore, no difference was observed for CD161, CD57 and HLA-DR expression on CD56+CD3− or CD56+CD3+ cells between the 3 groups (data not shown). No significant difference was observed for CD57 expression in remission compared to controls (p = 0.986). No significant difference was seen in the expression of HLA-DR on CD3+CD56− cells in remission (6.48±1.24%) compared to active RA (4.49±0.53% (p = 0.406)) or controls (7.40±1.77% (p = 0.873)). Furthermore, no difference was observed for CD161, CD57 and HLA-DR expression on CD56+CD3− or CD56+CD3+ cells between the 3 cohorts.

### CD94 and NKG2A expression on CD3+CD56− cells

**Figure 3:**
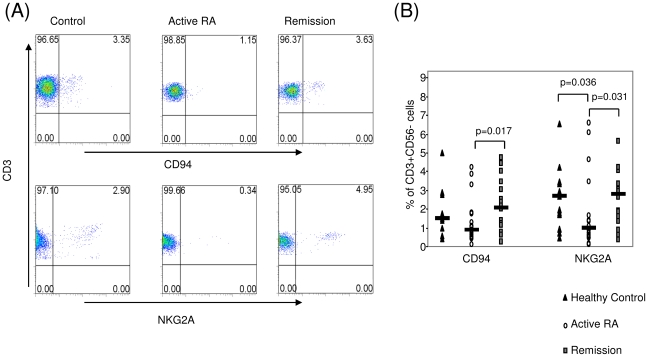
Expression of NKRs CD94 and NKG2A in active RA, Remission RA and healthy controls. (A) Representative dot plots of CD94 and NKG2A expression on healthy control, patient with active RA and patient in remission following TNF inhibitor therapy. (B) CD94 and NKG2A expression on CD3+CD56− cells in healthy controls (▴), patients with active RA (○) and patients in remission following TNF inhibitor therapy (▪). *p<0.05 significantly different.

A significant increase in CD94 expression was found in remission (2.16±0.31%) compared to active RA (1.33±0.29%) (p = 0.017). This was accompanied by a significant increase in the expression of the inhibitory receptor, NKG2A, in remission (2.67±0.37%) compared to active RA (1.76±0.48%) (p = 0.031) (Figure 3A,B). Furthermore, NKG2A and CD94 expression was similar in remission compared to controls (2.61±0.39%) (p = 0.957) (Figure 3). No significant differences were observed for expression of the inhibitory KIR, CD158a and CD158b, in remission (0.09±0.02% and 0.93±0.32% respectively), active RA (0.05±0.01% and 0.66±0.14%) or controls (0.14±0.05% and 1.12±0.64%). Finally, we observed no difference in CD94 and NKG2A expression on CD56+CD3− or CD56+CD3+ populations between the 3 cohorts (data not shown).

### T cell populations in patients maintaining or withdrawing TNFi

**Figure 4:**
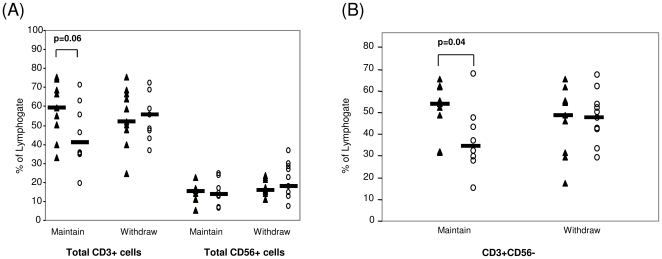
Total CD3+, Total CD56+ and CD3+CD56− T cell populations in patients maintaining or withdrawing therapy. Graphs illustrate total CD3+, Total CD56+ and CD3+CD56− T cell populations (expressed as a percentage of the lymphogate) in patients maintaining or withdrawing therapy at baseline (▴) and three months (○). *p<0.05 significantly different.

Total numbers of CD3+ cells significantly decreased from 58.05±4.98% to 44.16±5.75% in patients maintained on therapy (p = 0.06) (Figure 4A), in contrast to total CD56+ NK cells where no difference was observed (Figure 4A). CD3+CD56− T cells also decreased from 51.20±4.11% to 37.53±5.24% (p = 0.04)) in patients maintaining therapy (Figure 4B). No significant difference was observed between the frequency of the CD3+ population of lymphocytes at baseline (54.33±4.36%) compared to the three month time-point (56.06±3.57%) in the patient cohort withdrawing therapy (p = 0.78).

### CD94 and NKG2A expression in patients maintaining or withdrawing TNFi

**Figure 5:**
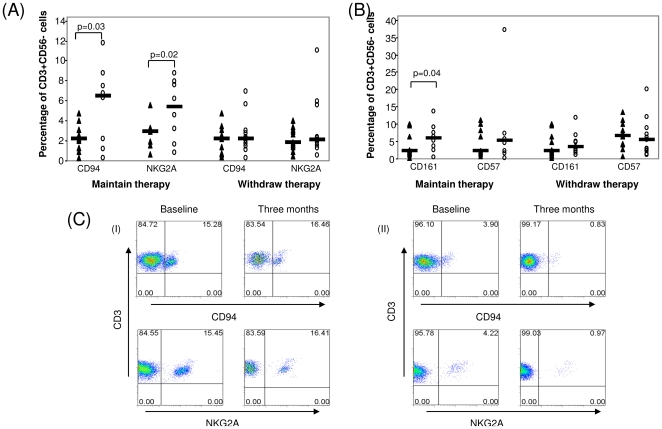
Expression of NKRs CD94, NKG2A, CD161 and CD57 in patients maintaining or withdrawing therapy. Graphs illustrate (**A**) CD94 and NKG2A expression and (**B**) CD161 and CD57 expression at baseline (▴) and three months (○) in patients maintaining or withdrawing TNF inhibitor therapy. (C) Representative dot plots of two patients withdrawing TNF inhibitor therapy. Patient (I) remained in clinical remission for the study duration and maintained expansion of CD94 and NKG2A; Patient (II) had a flare in disease activity 6 months after withdrawal of therapy with loss of expansion of CD94 and NKG2A at 3 months (i.e. prior to clinical relapse). *p<0.05 significantly different.

A significant increase in CD94 expression on CD3+CD56− T cells at three months (5.59±1.34%) compared to baseline (2.33±0.48%) was demonstrated in patients maintaining TNFi (p = 0.03). This was accompanied by a significant increase in CD3+CD56− expression of inhibitory receptor NKG2A (2.74±0.49% at baseline compared to 5.59±1.34% at 3 months (p = 0.02)) (Figure 5A). No difference was observed in expression of the inhibitory KIR, CD158a or CD158b, in either cohort between baseline and three months (data not shown). Figure 5C shows representative flow cytometry dot plots from 2 patients who withdrew TNFi. Patient 1 maintained remission throughout the study period. No change was observed in CD94 or NKG2A expression between baseline and three months in this patient; whereas Patient 2 had a flare in disease activity (DAS28>2.6) at six months. A reduction in the expression of CD94 and NKG2A on CD3+CD56− cells was demonstrated at the three month follow-up prior to clinical evidence of a flare in disease activity, suggesting reduced expression of CD94/NKG2A may be associated with RA flare.

CD161 expression was significantly higher in the group maintaining therapy at 3 months (6.08±1.39%) compared to baseline (3.97±1.24%) (p = 0.04). CD57 expression was also higher at 3 months (8.39±3.98%) compared to baseline (4.83±1.40%) but this did not reach statistical significance. (Figure 5B). We did not observe changes in the expression of CD161 or CD57 by the CD3+CD56− population of lymphocytes at three months in the patient cohort withdrawing TNFi (Figure 5B).

### Loss of CD94/ NKG2A expression is associated with flare in disease activity after withdrawal of TNFi

**Figure 6:**
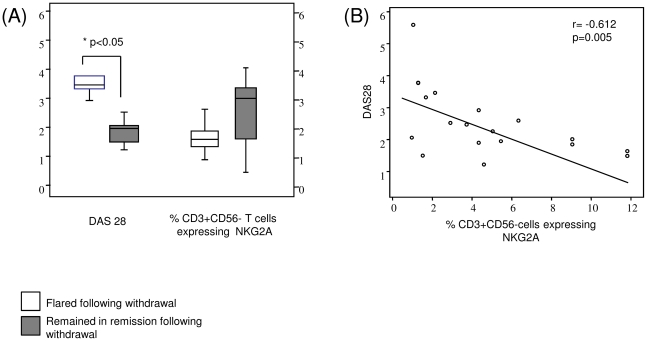
Associations between clinical disease activity and NKG2A expression. (A) Flare in disease activity (Increased DAS28) is associated with reduction in NKG2A expression on CD3+CD56− cells. (B) Negative correlation between DAS28 and T cell expression of *p<0.05 significantly different.

Figure 6A demonstrates DAS28 and NKG2A expression by T cells in patients who withdrew from or remained on TNFi. Open boxes represents patients who flared and grey boxes represents patients who stayed in remission. DAS28 is significantly higher in patients who flared which is associated with a parallel reduction in expression of NKG2A. Figure 6B demonstrates a negative correlation between DAS28 at six months follow-up and baseline NKG2A expression (r  = −0.612; p = 0.005).

## Discussion

The development of TNFi has revolutionised the treatment of inflammatory arthritis including RA leading to clinical, laboratory and functional improvement [3]–[5]. Initially prescribing TNFi was limited to patients who had failed a number of DMARDs, however recent data has shown that early initiation of therapy may reduce disability and future disease burden [3], [24], [25]. Recent guidelines suggest treatment of RA to a target of remission (or low disease activity in patients with established disease) with regular review to allow escalation of therapy as necessary to reach these targets. Therefore, potentially more patients will be prescribed biologic therapies at an earlier stage in the disease process [26]. Limitations of early treatment include the costs involved in treating patients with expensive medication for long time-periods, increasing the financial burden on already over-stretched healthcare systems. Furthermore, up to 30% of patients fail to respond to TNFi, therefore prediction of response is an important focus of research.

In this study, we identified markers in peripheral blood that differentiate active RA patients from patients in remission. While no difference was observed in the frequency of the CD3+CD56− populations between the three cohorts, expression of CD161, CD57 and CD94/NKG2A by T cells was significantly increased in the remission cohort versus active disease. Although CD4^+^CD28^−^ cells are contained within this cell subset they were not specifically assessed in the study. Following withdrawal of TNFi, the expression of CD94 and NKG2A by T cells was significantly increased in patients who remained in remission versus patients whose disease flared. Finally, an inverse relationship between the expression of NKG2A by T cells and DAS28 was demonstrated. Therefore, specific populations of NKR+ lymphocytes in the circulation of RA patients is associated with response to therapy and remission, and may be linked to disease flare following withdrawal of TNFi, prior to clinical symptoms of disease.

Approximately 30% of patients commencing on TNFi will enter clinical remission (defined as DAS28<2.6). In our cohort of patients, 45.45% flared within 6 months of stopping therapy despite commencing optimal DMARDs at the time of cessation of TNFi. Only one patient required recommencement of therapy after flare with the other patients reaching the 6-month endpoint off biologic therapy. Optimal DMARD therapy was commenced at TNFi withdrawal as it has previously been demonstrated that patients with established RA ceasing TNFi will flare following cessation of therapy with median time to flare between 13.5 and 15 weeks [27].

Commencing TNFi at the time of diagnosis of RA may allow for remission induction with 70% of patients maintaining response two years after withdrawal of infliximab therapy [28]. The BeST study also demonstrated that early intervention with TNFi could allow for withdrawal after a defined period, however a proportion of patients in the infliximab/DMARD group required continuation of biologic therapy [29], [30]. Similar remission rates have been demonstrated in the early commencement of other commercially available TNFi. In clinical practice patients commencing on therapy are maintained indefinitely on treatment once a clinical response is achieved and the treatment is well tolerated. Patients in clinical remission are also treated indefinitely and it is not clear if increasing time between infusion or injection or institution of ‘drug holidays’ will be tolerated. The duration of potential drug holidays is also unclear. RA is a heterogeneous disease and it is logical that some patients would tolerate longer drug holidays than others.

The effect of TNFi on NK receptor expression on T cells in RA has not been systematically studied. A significant increase in T cells expressing the NK receptors CD161, CD57, CD94/NKG2A was observed in remission compared to active RA. The expansion of CD161 was maintained in patients following withdrawal of therapy however there was significant reduction in CD94/NKG2A in patients who flared.

CD94 in association with NKG2A delivers inhibitory signals to T cells recognising HLA-E. CD94/NKG2A expression which was elevated in patients in remission compared to those patients with active disease, suggests this molecule plays a key role in the regulation of inflammation in RA. Supporting this hypothesis, it has been shown that CD94/NKG2A plays a key role in the regulation of pro-inflammatory events in RA [31]. IFNγ and TNFα production were markedly enhanced by antibody masking of the CD94/NKG2A receptor *in vitro*, suggesting that CD94/NKG2A is a key regulator of pro-inflammatory cytokine production [31] **.** A significant reduction in circulating lymphocytes expressing CD94/NKG2A was demonstrated in patients with new onset of psoriasis [32]. In addition, other studies have examined the effect of targeting NKG2A+ NK cells in a mouse model of autoimmune encephalomyelitis. Treatment of mice with a CD94/NKG2A/C/E specific F(ab’)2 fragment enhanced NK cell mediated elimination of activated autoreactive T cells resulting in inhibition of inflammatory damage to spinal cord which was associated with decreased infiltration of T cells [33]. Together these suggest that CD94/NKG2A maybe an important molecule involved in regulation of inflammation in a number of different autoimmune diseases by different mechanisms.

We observed a significant increase in the expression of CD161 by T cells in patients in remission. CD161 is a C-type lectin receptor, but little is known about its function in autoimmune diseases. CD161 and CD57 can both be expressed by NKT cells and are associated with sub-population of CD4+ T cells [34], [35]. Increased frequencies of CD4+CD28− expressing CD161 and CD57 in patients with active RA have been demonstrated [21], [36]–[38], and CD4+CD161+ T cells preferentially accumulated at the outer edge of lymphoid aggregates [38], while CD57 expression may render cells more susceptible to activation-induced cell death by apoptosis [39].

Recent studies have shown that sub-populations of CD4+ T-cells that co-express CD161 and CCR6 are potent producers of IL-17 suggesting CD161 is an important marker for human T-helper cells producing IL-17 (Th17) [40]. While its precise role is unclear, studies suggest that CD161 may regulate T cell adhesion, transendothelial cell migration and invasion [41]–[43]. Consistent with our results, studies have also shown a reduction in CD161+ T cells in the inflamed mucosa of ulcerative colitis compared normal control colon [44], and in psoriasis patients persistence of CD161 expression was demonstrated in plaques while lesional T-cell subset were reduced following 16 weeks of therapy [45]. Furthermore, CD161 can partially inhibit TNFα production by CD8+ T cells and therefore it may play a role in maintaining remission in patients following withdrawal of TNFi [46]. CD57+ cells have also been implicated in the pathogenesis of RA through secretion of pro-inflammatory cytokines [20], [35], however some studies have shown that CD57+ T cells from healthy subjects suppress neutrophil development *in vitro* an effect that is impaired in RA patients with Felty's Disease and large granular lymphocyte syndromes [47]. We also demonstrated a significantly higher frequency of CD57^bright^ T cells in patients in remission compared to active RA, however expression did not change when disease flares following TNFi withdrawal. CD57^bright^ T cell express high levels of cytolytic enzymes, have reduced proliferative rates, are thought to be susceptible to activation-induced cell death and may represent a functional ‘exhausted’ subset of T cells [48]–[50]. Although we observed a consistent increase of CD161 and CD57 expression by the T cells of the RA patients in remission, the exact role CD161 and CD57 play in the pathogenesis and regulation of inflammation in RA remains to be determined.

In conclusion, we present a small, but comprehensive dataset demonstrating differences in CD94/NKG2A expression by T cells in patients' circulation measured at regular intervals following therapy withdrawal and thus may precede disease flares in disease activity, allowing the timely reinstitution of therapy. This may allow for drug holidays in patients with RA in clinical remission decreasing the economic burden of biologic therapies and allowing reassurance to patients of a reduced likelihood of clinical flare in disease activity.
